# DEUTERIUM TRANSFER ANALYSIS INCLUDING FOOD CHAIN FROM SEAWATER INTO ABALONE

**DOI:** 10.1093/rpd/ncac072

**Published:** 2022-09-09

**Authors:** Toshihiro Shibata, Yoshio Ishikawa

**Affiliations:** Department of Radioecology, Institute for Environmental Sciences, 1-7 Ienomae, Obuchi, Rokkasho, Kamikita, Aomori 039-3212, Japan; Tokyo Electric Power Company Holdings, Inc. Fukushima Daiichi Decontamination & Decommissioning Engineering Company, Release and Environmental Monitoring Group, 1-1-3 Uchisaiwai-cho, Chiyoda-ku, Tokyo 100-8560, Japan; Tokyo Electric Power Company; Department of Radioecology, Institute for Environmental Sciences, 1-7 Ienomae, Obuchi, Rokkasho, Kamikita, Aomori 039-3212, Japan

## Abstract

Tritium is released into the ocean from nuclear facilities located at coastal areas. In addition, tritiated water is decided to be released into the ocean from the Fukushima Dai-ichi Nuclear Power Plant. Although released tritium concentration would be strictly controlled, impact of tritium on the marine products is major concern for the public. In this study, deuterium transfers from seawater into seaweed (ulva) and abalone were measured. In addition, organically bound deuterium (OBD) transfer from ulva into abalone was measured. OBD concentrations in ulva were saturated in 2 weeks and those in abalone were saturated in 6 months. Ulva and abalone were exposed to seawater containing 0.2% (mol-D/mol-H) deuterium. Maximum OBD concentrations in ulva were ~0.1% (mol-D/mol-H) and those in abalone muscle were ~0.035% (mol-D/mol-H). Numerical deuterium transfer model was constructed. Obtained numerical model well represented the OBD-enriched ulva feeding experiment.

## INTRODUCTION

Tritium is released into the ocean from various nuclear facilities located at coastal areas, especially from CANDU reactors and fuel recycling plants. In addition, coolant water, whose radiation pollutants except for tritium are removed by multi-nuclide removal facility (the Advanced Liquid Processing System (ALPS)), is decided to be released as ALPS-treated water into the ocean from the Fukushima Dai-ichi Nuclear Power Plant. Although released tritium concentration would be strictly controlled and will not cause significant radiation dose, impact of tritium on the marine products is major concern for the public. Therefore, analyzing tritium behavior and assessing actual time-dependent tritium concentration are important issues. When tritium is released into hydrological environment as waste liquid, most of tritium would be released as HTO. Tritiated water is converted to organically bound tritium (OBT) by photosynthesis in plants^(1, 2)^. Because OBT has longer biological half lifetime and larger impact than HTO, analyzing the behavior of OBT is important^(3)^.

Previously, tritium transfer from water, including seawater into shellfish or plants, is investigated^(4–6)^. Tritium behavior in the environment is affected by various parameters^(7,8)^. Especially, feeding rate and tritium concentration in food materials would be important factors to construct a numerical model to estimate the tritium concentration in animals. However, it is not easy to estimate the feeding rate of planktotrophic fishes or shell fishes. In this study, deuterium transfer from seawater into abalone (*Haliotis discus hannai*), which is seaweed feeder, including deuterium transfer via seaweed, was analyzed in order to construct a dynamic deuterium transfer model from seawater into shellfish. Because annual usage of tritium is controlled by the low, deuterium was used in this study. In addition, deuterium transfer from seawater into seaweed organically bound deuterium (OBD) was measured to obtain transfer coefficient. Abalone and ulva (*Ulva prolifera*) were exposed to deuterium-enriched seawater to observe deuterium transfer from seawater into OBD in ulva and abalone. Then, deuterium transfer from ulva OBD into abalone OBD was analyzed. Finally, tritium transfer model from seawater into abalone, including food chain within three compartments, was constructed.

## MATERIALS AND METHOD

### Ulva exposure experiment to deuterium

Ulva was collected from the Mutsu bay, Aomori, Japan. The 3-cm diameter ulva discs (~100 mg) were clipped from the collected ulva body. Clipped ulva discs were cultivated in seawater containing 0.2% (mol D/mol H) deuterium at 15°C for 3 weeks. Light–dark cycle was set to 12-h light period and 12-h dark period. Three pieces of ulva were collected at each sampling period. Non-exchangeable OBD (Nx-OBD) concentration in ulva was measured by the following method.

### Abalone exposure experiment to deuterium

Abalone was purchased from aqua culturist in Ehime prefecture, Japan (~6 cm (shell length), 2 y old). They were cultivated in seawater containing 0.2% (mol D/mol H) deuterium for 300 d at 15°C in a 700-l tank without individual identification and area separation. Cultivation was started with 120 abalones. Approximately, 100 l of deuterium-enriched seawater was changed per 2 or 3 d (three times per a week). Approximately, 0.3 g (1% of averaged wet weight of abalone, including the shell) of assorted meal per an abalone for aquaculture with background deuterium concentration was fed one time per day (six times per a week). Five abalones were collected at each sampling after 3 d fasted. They were dissected to obtain hepatopancreas, including reproductive system and muscle (foot) tissues. Weight and Nx-OBD concentration in each organ were measured. Nx-OBD concentration was measured by the following method: 82 abalones were used to measure OBD concentration, and the others were dead during exposure. Details about the weight and size of abalone are shown in supplemental data.

### Measurement of deuterium concentration

OBD concentrations in abalone and ulva were measured by the following method. Collected samples were lyophilized and powderized. 0.2 g of lyophilized samples were stirred for 24 h in 10 ml of deuterium depleted water at 4°C and lyophilized for three times to remove exchangeable OBD,^9^ such as deuterium on hydroxyl group or amino group. Then, deuterium concentration was measured by elemental analyzer (vario PORO cube, Elmentar) and mass spectroscopy (IsoPrime 100, Isoprime).

Deuterium concentration in cultivating water was measured by gas chromatography system (GC-6890, Agilent Technologies). Porapak-Q (80/100 mesh, 1/8 in × 6 ft) was used as pre-column and Molecular Sieve 13X (45/60 mesh, 1/8 in × 10 ft) was used as analysis column. Hydrogen gas was used for carrier gas. Deuterium concentration in cultivating water was monitored one time per week. It was checked against and adjusted to designed concentration.

### OBD feeding experiment

In order to measure OBD transfer rate from foods into abalone, deuterium-enriched ulva was fed to abalone. Deuterium-enriched ulva was obtained by 60 d of exposure to deuterium-enriched seawater (0.2% (mol D/mol H)) at 20°C under 24 h lighting. Approximately, 200 discs of ulva were collected in an experiment and Nx-OBD concentrations in five pieces of them were measured. Deuterium concentrations in this ulva discs were assumed to be represented by averaged deuterium concentration in the measured five samples. Remained ulva discs were dried and fed to abalone for 24 or 30 d. Each abalone was kept in plastic cage in 300-l seawater tank. Three days after feeding finished, abalones were collected. Then, their weight of each organ and Nx-OBD concentration in hepatopancreas and muscle were measured. experiments were performed for three times, and 6–11 abalones were used for one experiment.

### Deuterium transfer model

**Figure 1:**
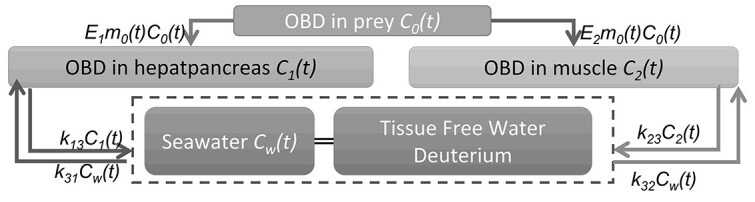
Outline of analytical model between seawater and abalone.

Experimental data were analyzed by a numerical model based on compartment model, as shown in Figure 1. Previous study presented numerical model for freshwater bivalve constructed with six compartments^(4)^. However, in this study, simplified numerical model with four compartments was constructed. Because water exchange between tissue-free water and rearing water is much faster than the experiment time, tissue-free water of each organ and rearing water are assumed to be in equilibrium and are described as one compartment. In addition, organically bound hydrogen, including D and T, was assumed to be homogeneously distributed in each organ, and one compartment of organically bound hydrogen was defined for each organ because it is hard to identify slowly exchangeable material and faster one and to separate between each other.

Deuterium transfer between hepatopancreas and muscle was not considered. Hydrogen transfer, including both D and^1^H, from foods was considered. Deuterium concentration in tissue-free water was assumed to be in equilibrium with deuterium in seawater as described above. Deuterium intake rates from seawater to abalone each organ OBD were assumed to be equal to excrete rate. Ingested materials are assimilated in digestive organ and hepatopancreas. Then, they are distributed into each part of body. However, assimilation process and assimilated material transfer process would be too fast to measure in the experiment in this study (abalones were dissected 3 d after the last feeding). In addition, time-depending deuterium concentration in the circulatory system of abalone cannot be observed in our experimental system. Therefore, in this model, it was assumed that the ingested deuterium is delivered to each part of body directly depending on the partition coefficient (*E*_1_ and *E*_2_). Deuterium behavior in ulva and abalone were defined as (1), (2) and (3). Each parameter was fitted to deuterium exposure experiment data by least-square method.

(1) \begin{equation*} \frac{d{C}_a}{dt}={k}_a{C}_w(t)-{k}_b{C}_a(t)\end{equation*}

where *C_a_*(*t*) is the Nx-OBD concentration in ulva, *C_w_*(*t*) is the deuterium concentration in seawater, *k_a_* (d^−1^) is deuterium transfer coefficient from seawater into ulva and *k_b_* (d^−1^) is that from ulva into seawater.

(2) \begin{align*} \frac{d{C}_1(t)}{dt}=&\frac{E_1{C}_0(t){m}_0(t) dt+{M}_1{C}_1}{\left({E}_1{m}_0(t)+{M}_2\right) dt}\nonumber\\ &+{k}_{31}{C}_w(t)-{k}_{13}{C}_{1}(t) \end{align*}

(3) \begin{align*} \frac{d{C}_2(t)}{dt}=&\frac{E_2{C}_0(t){m}_0(t) dt+{M}_1{C}_2}{\left({E}_2{m}_0(t)+{M}_2\right) dt}\nonumber\\ &+{k}_{32}{C}_w(t)-{k}_{23}{C}_{2}(t) \end{align*}

where *C*_0_(*t*), *C*_1_(*t*) and *C*_2_(*t*) (mol D mol H^−1^) are Nx-OBD concentration in prey (ulva) abalone hepatopancreas and muscle, *C_w_*(*t*) (mol D mol H^−1^) is deuterium concentration in seawater, *E*_1_ and *E*_2_ are partition constants from prey to hepatopancreas and to muscle, *k*_31_ (d^−1^) is the deuterium transfer coefficient from seawater into abalone hepatopancreas and *k*_13_ (d^−1^) is that from hepatopancreas into seawater, *k*_32_ (d^−1^) is the deuterium transfer coefficient from seawater into abalone muscle and *k*_23_ (d^−1^) is that from muscle into seawater, *m*_0_(*t*) (mol d^−1^) is the hydrogen ingestion rate and *M*_1_ and *M*_2_ (mol) are amounts of hydrogen in hepatopancreas and muscle.

## RESULTS

**Figure 2:**
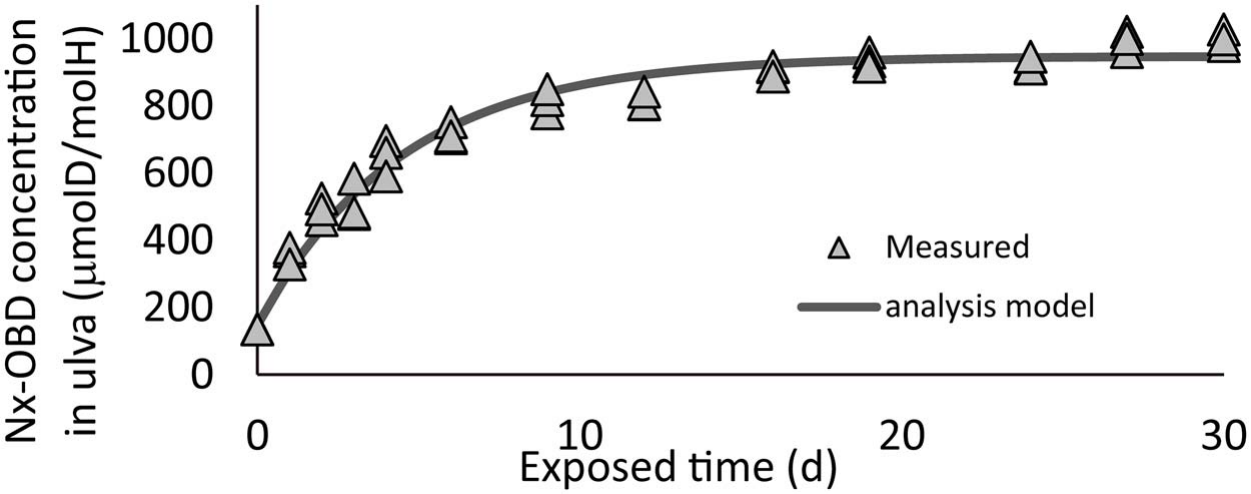
Time-dependent Nx-OBD concentration in ulva.

**Figure 3:**
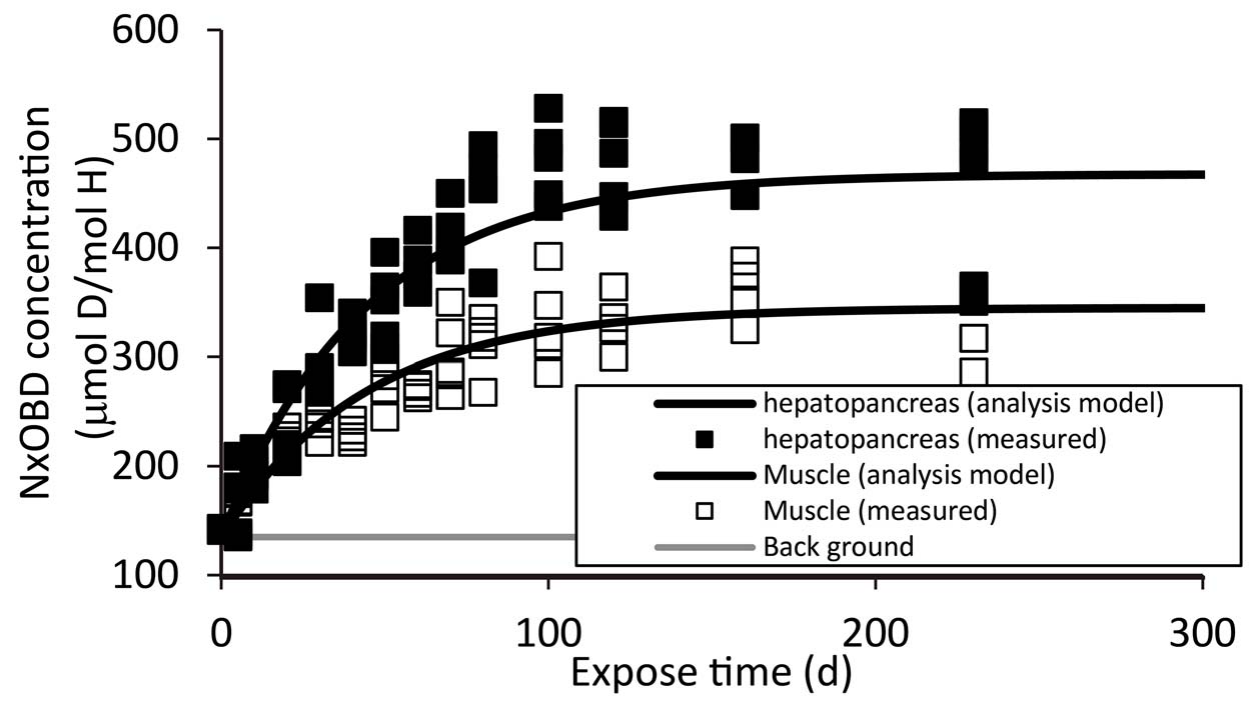
Time-dependent Nx-OBD concentration in abalone; background Nx-OBD concentration is indicated by short dashed line.

Measured Nx-OBD concentrations in ulva and abalone exposed to deuterium-enriched seawater are shown in Figures 2 and 3. Nx-OBD concentrations in ulva and in abalone were almost saturated during experimental period. Observed maximum Nx-OBD concentration in ulva was ~0.1% (mol D/mol H) until 3 weeks exposure. Nx-OBD concentration in abalone hepatopancreas and muscle have increased for 150 d up to 0.05% (mol D/mol H) in hepatopancreas and to 0.04% (mol D/mol H).

Averaged Nx-OBD concentration in prey ulva used for Nx-OBD feeding experiment was 0.59% (mol D/mol H). Averaged feed dosage, dry weight of muscle and hepatopancreas are shown in Table 1. Observed Nx-OBD concentration in hepatopancreas was from 0.015 to 0.055% (mol D/mol H) and that in muscle was approximately from 0.015 to 0.025% (mol D/mol H).

**Table 1:** Experimental time, averaged Nx-OBD concentration in fed ulva, feed dosage, dry weight of muscle and hepatopancreas for each feeding experiment

Experiment set	Experiment time (d)	Prey ulva Nx-OBD concentration (mol D mol H^−1^)	feed dosage (g-dry d^−1^)	hepatopancreas weight (g-dry)	muscle weight (g-dry)
(1)	24	5.9E-4 ± 4.2E-5	5.1E-2 ± 2.8E-2	2.7E-1 ± 2.2E-2	8.4E-1 ± 1.1E-1
(2)	30	6.2E-4 ± 9.1E-5	1.0E-1 ± 5.5E-2	1.1 ± 2.9E-1	3.1 ± 4.8E-1
(3)	24	5.9 E-4 ± 4.2E-5	3.3E-2 ± 1.7E-2	6.5E-1 ± 1.7E-1	2.7 ± 5.2E-1

Estimated parameters in numerical tritium transfer model (as given in Equations (1–3)) are shown in Table 2. In order to estimate parameters, it was assumed that each abalone was fed 0.3 g of assorted meal in each time and their dry weight of muscle and hepatopancreas were 0.9 and 3.2 g during the deuterium exposure experiment. The growth of abalone during experiment was not considered. Averaged whole wet weight, including the shell, dry weight of muscle and hepatopancreas, of all samples were 30, 3 and 0.9 g at the time of sampling. Time-dependent OBD concentrations in ulva, abalone hepatopancreas and muscle during deuterium exposure experiment calculated with the obtained parameters are shown in Figures 2 and 3.

**Table 2:** Obtained numerical model parameters

*E*_1_	*E*_2_	*k*_31_ = *k*_13_ (d^−1^)
1.2E-1 ± 1.6E-2	3.6E-1 ± 5.0E-2	4.0 E-3 ± 4.2E-4
*k*_32_ = *k*_23_ (d^−1^)	*k_a_* (d^−1^)	*k_b_* (d^−1^)
2.5 E-3 ± 3.0E-4	2.3E-1 ± 1.3E-2	9.3E-2 ± 4.6E-3

**Figure 4:**
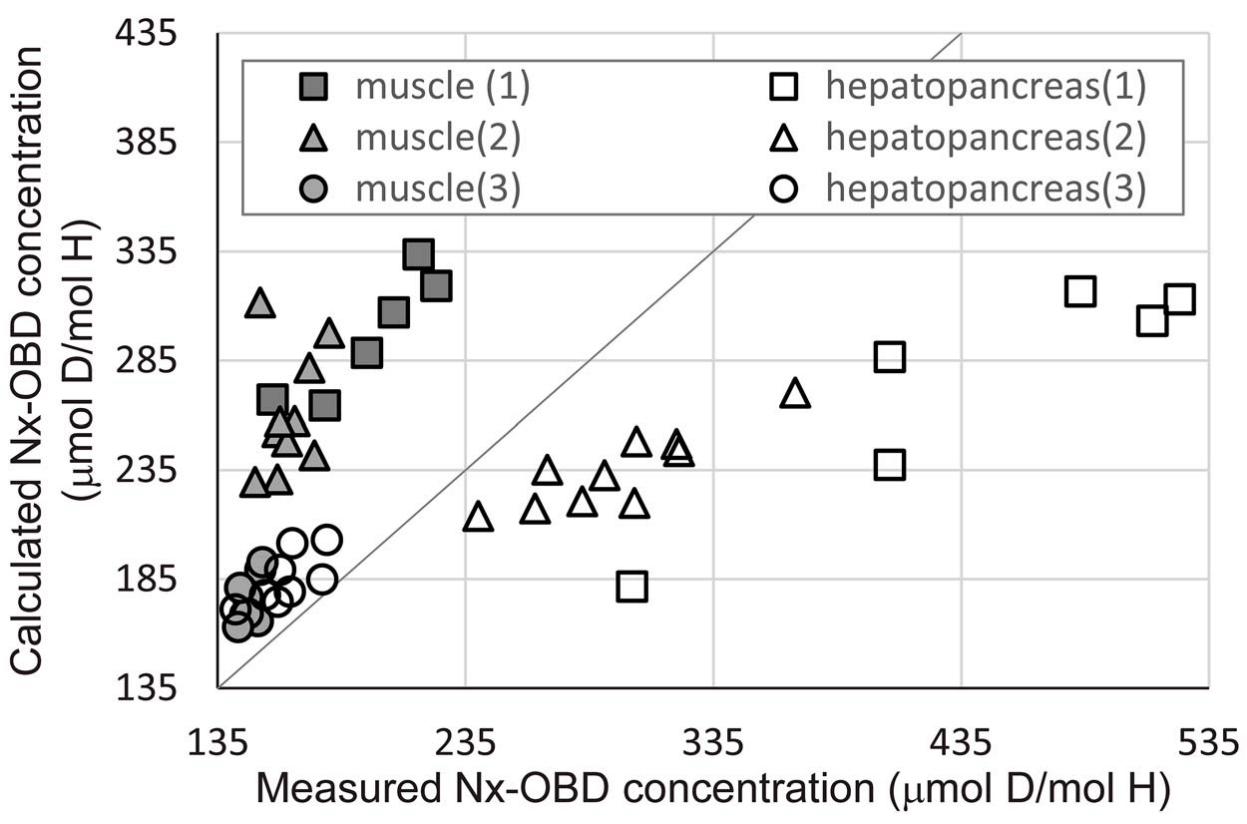
Correlation plot of measured Nx-OBD concentration in hepatopancreas and in muscle after feeding experiment and those of estimated; feeding experiment was performed three times

Deuterium concentration after OBD feeding experiment was calculated with obtained parameters, measured weight of each abalone and actual feeding amount. Correlation plot of experimental data of feeding experiment and estimated value is shown in Figure 4.

## DISCUSSION

Time-dependent Nx-OBD concentration was well described by the analysis model shown in Figures 2 and 3. Increasing rate of Nx-OBD concentration in abalone muscle was smaller than that in hepatopancreas. Estimated saturated deuterium concentration in abalone muscle was ~20% lower than that in hepatopancreas. These would be caused differences of metabolism rate between hepatopancreas and muscle.

Deuterium intake and release coefficient of ulva was not equal. Saturated Nx-OBD concentration in ulva was 50% of seawater as previously reported^(6,7)^. One of the considerable reasons why intake rate coefficient and release rate coefficient of ulva were not equal to each other would be due to the isotopic effect in the photosynthesis process.

Previous studies have shown that OBT concentration in aquatic biota is lower than HTO concentration in environment water^(4–6)^. When food material was not labeled, OBT concentration in organisms can be <50% of that in water^(6)^. Nx-OBD concentration in abalone exposed to deuterium-enriched water (0.2% (mol D/mol H)) was <0.06% (mol D/mol H) and 30% of seawater deuterium concentration in this study.

Calculated Nx-OBD concentration in abalone after OBD feeding experiment was 0.5–1.5 times of measured value. Estimated Nx-OBD concentration in muscle after the OBD feeding experiment tended to be overestimated, as shown in Figure 4. It was ~10% higher than the measured value. This could be because the experiment period of OBD feeding experiment was much shorter than that of deuterium-enriched seawater exposure. Estimated Nx-OBD concentration in hepatopancreas after OBD feeding experiment tended to be over- or underestimated depending on the experiment group, as shown in Figure 4. When abalones ate smaller amount of ulva, measured Nx-OBD concentration in hepatopancreas was higher than the estimated value, and when they ate larger amount, measured Nx-OBD concentration was lower than the estimated value. During OBD feeding experiment, deuterium concentration in seawater was kept to almost background level. One considerable reason why these differences are caused is due to the differences of the activities of each abalone. It can be considered that abalones which eat larger amount of food would tend to be more active and tend to require larger amount of energy. Then, ingested foods may be used to generate energy, and transfer rate from foods to abalone body may be decreased. Therefore, under- or overestimation could be caused when deuterium behavior is calculated by the constant partition constant (*E*_1_ and *E*_2_). However, it would be hard to observe and to simulate the activity of abalones in the natural environment. Therefore, some amount of estimation error in Nx-OBD concentration in abalone, especially, in hepatopancreas is not avoidable.

## CONCLUSION

Deuterium transfer rates from seawater into ulva and abalone were analyzed by the compartment model. This model can describe tritium transfer from seawater into ulva and abalone, including OBT transfer from seaweed into abalone, with small adjustment. When time-dependent tritium distribution in inshore area is provided, this model will enable to estimate tritium concentration in seaweeds and abalone.

## Supplementary Material

supplemental_data1_ncac072
